# Current and Future Development in Lung Cancer Diagnosis

**DOI:** 10.3390/ijms22168661

**Published:** 2021-08-12

**Authors:** Reem Nooreldeen, Horacio Bach

**Affiliations:** Division of Infectious Diseases, Faculty of Medicine, The University of British Columbia, Vancouver, BC V6H 3Z6, Canada; reemnooreldeen@gmail.com

**Keywords:** lung cancer, diagnosis, imaging, biomarkers, predictors, body fluids

## Abstract

Lung cancer is the leading cause of cancer-related deaths in North America and other developed countries. One of the reasons lung cancer is at the top of the list is that it is often not diagnosed until the cancer is at an advanced stage. Thus, the earliest diagnosis of lung cancer is crucial, especially in screening high-risk populations, such as smokers, exposure to fumes, oil fields, toxic occupational places, etc. Based on the current knowledge, it looks that there is an urgent need to identify novel biomarkers. The current diagnosis of lung cancer includes different types of imaging complemented with pathological assessment of biopsies, but these techniques can still not detect early lung cancer developments. In this review, we described the advantages and disadvantages of current methods used in diagnosing lung cancer, and we provide an analysis of the potential use of body fluids as carriers of biomarkers as predictors of cancer development and progression.

## 1. Introduction

Lung cancer is the most common cause of cancer-related deaths in North America and other developed countries. According to the 2020 special report on lung cancer, this disease is the most commonly diagnosed cancer and the leading cause of cancer death in Canada [1]. The impact imposed is highlighted by statistics reporting a higher number of Canadians dying of lung cancer than colorectal, pancreatic, and breast cancers combined. For instance, approximately 30,000 Canadians will be diagnosed with lung cancer, with a projection of 21,000 death in 2020. Globally, the cancer burden is projected to double by 2050, with lung cancer at the top of the list [1].

People die from lung cancer because it is often not diagnosed until the cancer is at an advanced stage. Detailed pathogenesis, effective early detection, and suitable drugs help in the effective therapy of lung cancer. Thus, the earliest diagnosis of lung cancer is crucial, especially in screening high-risk populations (e.g., smokers, exposure to fumes, oil fields, toxic occupational places, etc.) with an urgent need to identify novel biomarkers. Furthermore, accurate diagnosis is vital for the most suitable treatment of individual patients with lung cancer. Thus, there is an urgent need to identify sensitive and specific biomarkers for early diagnosis.

Currently, low-dose CT (LDCT) is routinely used for lung cancer screening. In addition, a trial (NELSON) has shown that this particular screening has a selectivity of 85% and a specificity of 99% compared to no screening [2]. A recent study showed that the overall false-positive rate reached 81% [3]. This very high number required additional imaging or testing to confirm the results.

This review will focus on the current screenings for early diagnosis of lung cancer, the use of potential biomarkers, and the current state of liquid biopsy-based approaches for screening will be discussed as well.

## 2. Lung Cancer Staging

To understand the timing of the screening and the progress of lung cancer, a brief explanation of the staging follows.

Lung cancer is divided into two major groups, the Small-Cell Lung Cancer (SCLC) and Non-Smal-Cell Lung Cancer (NSCLC).

SCLC stage. This is a central tumor arising from the airway submucosa as a peri-hilar mass. Histological studies found that this type of cancer originates from neuroendocrine cells of the basal bronchial epithelium [4]. The cells are small, spindle or round cells with scanty cytoplasm, granular chromatin, and the observation of necrosis is a common finding [5]. SCLC can be subtyped into pure or combined with NSCLC. This cancer is characterized because it may metastasize to the brain, liver, and bone [4] and is classified as limited or extensive stages [6].

The limited SCLC stage is confined to a single radiation point, the ipsilateral mediastinum, and the ipsilateral mediastinal or supraclavicular lymph nodes. In the supraclavicular lymph nodes, it is considered in that category as long it is present on the same side of the cancer chest.

On the other hand, the extensive SCLC is not limited to a single radiation point in the lung and metastasizes to the second lung lobe, lymph nodes, and other parts of the body, such as bone marrow.

NSCLC stage. This type of cancer is histologically divided into adenocarcinoma, large-cell carcinoma, and squamous cell carcinoma, and the stages categorized it. The nomenclature of the stages was formulated by the American Joint Committee on Cancer (AJCC) [7], and it is defined as the TNM staging system. The TNM system helps to determine the stage of cancer using the size of the primary tumor (T), the spread of the tumor to lymph nodes (N), and the presence of metastasis (M). Thus, the final TNM classification involves the combination of tumor characteristics (T) categorized as T1 to T4, the lymph nodes involved (N0-N3), and the presence (M1) or absence of metastasis (M0) (Table 1).

According to tumor characteristics, in the T2 category, atelectasis or incomplete lung inflation can be seen in part of the lung. The tumor could be found to invade the visceral pleura and the main bronchus more than 2 cm from the carina. This extends in the T3, where atelectasis develops to the whole lung. The tumor invades the phrenic nerve, diaphragm, chest wall, mediastinal pleura, and approaches closer to the main bronchus, less than 2 cm from the carina. Stage T4, or the invasion stage, is when the tumor invades mediastinal organs, vertebral bodies, and the lung carina.

Concerning lymph node involvement, defined as N0 to N3 (Table 1), lymph node involvement ranges from no lymph nodes to ipsilateral to contralateral involvement according to the stage.

Metastasis is only staged upon the presence of M1, where bilateral lesions, distant metastasis, and malignant pleural effusion could be seen. On the other hand, the absence of metastasis is defined as M0.

The main challenge imposed by lung cancer on public health is its poor prognosis because of the advanced stage, as most patients (>75%) have either a stage III or IV disease at diagnosis. Moreover, the prognosis of patients with lung cancer is strongly correlated to the disease stage. For example, whereas patients with clinical stage IA have a 5-year survival of about 60%, the clinical stage II-IV disease has a 5-year survival rate ranges from 40% to less than 5%.

Over two-thirds of the patients have regional lymph node involvement or distant disease at the time of presentation [8]. This is due to the lack of effective early detection strategies that allow for higher potential cure rates. In addition, lung cancer is commonly resistant to standard therapeutic modalities such as chemotherapy and radiation, which together with the lack of successful treatment for metastatic disease, adds to the dismal outcome [8].

Historically, the only diagnostic tests available for detecting lung cancer in its early stages were chest radiography and sputum cytology. However, results showed that these two screening methods failed in clinical trials and could not demonstrate their efficacy as mass screening tools [9]. Currently, screening for lung cancer with LDCT is recommended in high-risk populations defined as persons who are 55 to 74 years of age with a minimum smoking history of 30 pack-years or more (pack-years = number of cigarette packs smoked per day × the number of years smoked), who currently smoke, or have quit in the past 15 years and are disease-free at the time of screening [10]. Furthermore, recent developments in genomics have been used to define high-risk populations, making them more suitable for lung cancer screening for early diagnosis [11].

There is a body of knowledge related to predisposing factors and natural history for lung cancer that together with pathological findings can lead to the identification of cancer, but many questions are still open. It is well believed that identifying pre-invasive lesions might develop promising early intervention methods and a shift of therapeutic studies targeting only clinically verified lung cancer [11].

Lung cancer represents one of the most studied cancers regarding immunology. This is because lung cancer is driven by genetic and epigenetic aberrations explained by mutations affecting proto-oncogenes and tumor suppressors with the emergence of host immune deregulation. Advances in immune-genomic technologies have provided a platform for a better understanding of lung tumors on a molecular and genomic level. In this regard, genomic surveys of pre-malignant lung cancers have shed light on early alterations in their evolution, which allows the identification of therapeutic targets “tumor-type agnostic therapies” for early treatment and diagnosis [12].

## 3. Classification

As mentioned above, lung cancers are classified into two main histological types, SCLC and NSCLC [13]. SCLCs are aggressive lung tumors often caused by smoking and encompass 15–20% of all primary lung cancers. Interestingly, the gene amplification of the transcription regulators *MYC* is common in SCLC [14,15]. NSCLC can be divided into four subtypes: Lung adenocarcinoma (LUAD), lung squamous cell carcinoma (LUSC), large-cell carcinoma, and bronchial carcinoid tumor. Among these, LUAD is the most prevalent subtype of NSCLC and the most common primary lung tumor. LUAD frequently arises among female non-smokers, a category often missed on screening. It adopts a histologically glandular pattern with activating mutations affecting driver genes such as the oncogenes *KRAS* and *BRAF* and the epidermal growth factor receptor *EGFR* [12,13].

## 4. SCLC

This type of lung cancer is a poorly differentiated and high-grade neuroendocrine tumor, accounting for 10–15% of all lung cancers. SCLC is characterized by short tumor doubling time and metastasis at an early stage, with more than half of the patients diagnosed at an advanced stage. Therefore, screening and early diagnosis could lead to a better prognosis. The standard treatment for SCLC patients comprises chemotherapy combined with chest radiotherapy [16].

The immune system plays a crucial role in controlling tumor growth and progression via cancer immune surveillance, as suggested by current immune-oncology research. However, tumors can escape immune surveillance by inducing regulatory T cells to promote T cells’ dysfunctions and natural killer cells. This immunosuppressive state in patients with SCLC can influence their prognosis. Thus, aiming towards reversing the immunosuppressive status of SCLC provides hope for immunotherapy for SCLC patients. Preclinical and clinical trials on immune checkpoint inhibitors and adoptive cell therapy can introduce SCLC treatment modalities with a parallel investigation of new biomarkers to achieve precise treatment and early diagnosis [16].

Clinically, seven immune checkpoint inhibitor antibodies have been approved by the United States Food and Drug Administration (FDA) for the treatment of a variety of tumors: Ipilimumab that blocks cytotoxic T-lymphocyte antigen-4, and six antibodies that block PD-1/PD-L1 including pembrolizumab, nivolumab, atezolizumab, durvalumab, cemiplimab, and avelumab. These antibodies have achieved promising results in the treatment of recurrent SCLC. On the other hand, tumor vaccines, immunomodulators, cellular immunity, and other immunotherapy methods can play an increasingly important role in comprehensive tumor therapy. Reasonable treatment timing and optimal combined strategy are the hotspots of SCLC immunotherapy [17].

## 5. NSCLC

Over the last decade, tissue and/or blood biomarkers have helped guide patients’ treatment decisions with advanced NSCLC. Based on the detection of biomarkers, this has provided a channel for subgrouping patients. Evidence shows that treatment with targeted therapies has superior clinical outcomes than traditional cytotoxic chemotherapy [18,19].

Newly diagnosed NSCLC patients are opted to take biomarker testing for determining optimal treatment. Practical guidelines such as the CAP/IASLC/AMP, ASCO, and National Comprehensive Cancer Network guidelines help select the most appropriate biomarkers and assays to use [20].

There are many different types of tissue- and blood-based assays available for biomarker testing, all with their advantages and disadvantages that clinicians should understand when deciding which assays to use. For example, plasma-based assays have many advantages over tissue-based tests because the test is non-invasive, fast, and easily repeatable over time. Still, they may be less sensitive than tissue-based assays and cannot serve as stand-alone testing for patients with NSCLC. For example, the most common targetable mutation (*EGFR*) testing has been part of the standard practice since 2011.

NSCLC serves as a model for the successful application of “precision medicine” or the concept of using advanced genetic analysis of a patient’s tumor to obtain an individualized therapy plan in contrast with cancer treatment regimens assigned in a definite manner, based mainly on the organ of origin. The NCI-MATCH Trial (Molecular Analysis for Therapy Choice) is an example of an advanced precision medicine clinical trial, where genomic sequencing of a patient’s tumor is performed. The cancer treatment regimen is derived based on the genomic findings, not the organ in which cancer originated. Further advances in precision medicine depend on the development of novel diagnostic assays, which are needed to provide feedback (preferably quantitative) to oncologists regarding the efficacy of therapy [21].

Checkpoint immunotherapy (CPI) in metastatic NSCLC and the emergence of predictive biomarkers for CPI efficacy reinforces the importance of testing for both treatable genomic abnormalities and immune-related biomarkers. To this point, current national and international guidelines now recommend testing for alterations of the oncogenic targets *EGFR*, *ALK*, *ROS1*, *BRAF*, *RET*, *MET*, and *HER2*, along with immune biomarkers such as PD-L1 and tumor mutational burden (TMB) [22].

As highlighted above, both SCLC and NSCLC benefit from using biomarkers in early diagnosis and follow-up of a treatment and even choosing a treatment protocol.

## 6. Traditional Diagnosis and Screening

### 6.1. Screening High-Risk Groups

Screening of high-risk groups allows for early detection at a treatable and curable stage. As mentioned above, high-risk involves a history of heavy smoking (more than 30 pack-year), current smokers, or smokers who have quit less than 15 years ago, and between 55 and 80 years. Given that the American Cancer Society predicted 135,720 lung cancer deaths in 2020, more widespread screening could save 30,000–60,000 lives in the United States each year. The US Preventive Services Task Force USPSTF recommended lowering the starting age for screening from 55 to 50 years and the smoking history requirements from 30 to 20 pack-years. Medical providers have also recommended getting familiar with lung cancer screening guidelines and prescribing these exams for high-risk patients. Currently, only a fraction of the recommended population is screened [23,24,25].

### 6.2. Radiographic Screening and Diagnosis

In Japan, a study observed that annual clinic-based chest X-ray screening for lung cancer reduced 25% of lung cancer mortality for subjects screened annually [26]. Interestingly, results published from a study performed in Osaka, Japan, showed that high-risk smoker screening using low-dose helical computed tomography (LDCT) showed a reduction of 20% in lung cancer compared to standard radiographic screening [27].

In lung cancer diagnosis using chest radiography, the sensitivity for tumor detection is roughly 1 cm in diameter, which already has over 10^9^ cells with a potential of disrupted bronchial and vascular epithelia. CT is more effective in detecting peripheral lung lesions than plain radiography or conventional tomography of the whole lung. Spiral CT scans can continuously acquire data resulting in a shorter scanning time, a lower radiation exposure, and improved diagnostic accuracy than plain radiography. Then, this technique can image the whole chest in a very short time (one or two breath-holds), with a concomitant reduction in artifacts with a better outcome in missing nodules. Nodules as small as 1–5 mm can be shown with modern spiral CT technology. Lung cancer screening is now routinely done using CT, with or without additional adjunct tests such as sputum cytology. Two barriers that discourage its implementation for the general population are cost and accessibility. Furthermore, exposure to low-dose radiation increases the risk of a patient eventually developing breast, thyroid, or lung cancer, especially if they undergo multiple CT scans. LDCT may identify abnormalities that are not cancer (false positives) that require patients to undergo more invasive testing such as biopsies and surgery to remove the anomaly, presenting additional intra- and post-operative risks and complications [26,27].

Spiral CT scans have shown a better diagnostic ability for peripherally small tumor identification. However, the sensitivity of spiral CT for more centrally located tumors (primarily squamous cell carcinoma) is significantly lower than for peripherally located tumors [27]. Notably, approximately 40% of all participants recruited in the LDCT of the National Lung Screening Trial showed at least one positive screen, registering 96% false positives [28]. The high percentage of false-positive can be translated into costly screening and invasive procedures on smoking subjects free of lung cancer. Taken together, the screening of lung cancer using less expensive instruments and non-invasive techniques is a high priority in diagnosing lung cancer.

### 6.3. Sputum Examination

Another diagnostic procedure of lung cancer is the cytological examination of sputa, especially multiple samples, which helps detect central tumors from the larger bronchi (e.g., squamous- and small-cell carcinomas). In general, sputum samples failed to detect small adenocarcinomas (diameter ≤ 2 cm) that originated from the airway ramifications, such as small bronchi, bronchioles, and alveoli. This has become of greater importance because cigarette exposure changes (filters and decreased nicotine content) have increased adenocarcinomas and decreased squamous carcinomas. Sputum cytology’s sensitivity for early lung cancer is only in the 20–30% range from screening studies. Early studies showed that the ability to detect pre-malignant conditions depends on different factors such as the number and type of cells (deeper airways) [29]. Studies have also concluded that sputum cytology was insufficiently insensitive or accurate to be included in the routine workup of any patient suspected of having lung cancer [30].

Studies have shown that perhaps immunostaining could provide a more favorable outcome compared to sputum cytology. For example, an 8-year study at the Johns Hopkins hospital collected annual sputum specimens from individuals screened with known clinical outcomes. The sputum specimens were archived and screened for biomarkers that could indicate lung tumors in an early or pre-invasive stage [31]. As a result of this investigation, two monoclonal antibodies were studied to distinguish the pattern of marker expression. Results showed that positive staining with these antibodies predicted subsequent lung cancer approximately two years before clinical recognition of the disease based on chest X-ray and cytology. In addition, one of these antibodies (703D4) showed higher sensitivity and was later identified as recognizing the protein heterogeneous nuclear ribonucleoprotein (hnRNP) A2/B1 [32]. Following this study, the role of hnRNP A2/B1 overexpression for detecting pre-clinical lung cancer was studied in a large high-risk population, including 6000 Chinese tin miners who were heavy smokers and had an elevated lung cancer rate. This study indicated that the expression of hnRNP A2/B1 in epithelium cells from sputum was 2- to 3-fold more sensitive for early detection of lung cancer than standard chest X-ray and sputum cytology methods [31].

### 6.4. Bronchoscopy and Lung Tissue Biopsy

White light bronchoscopy (WLB) is the most commonly used diagnostic tool for obtaining a definite histological diagnosis of lung cancer. However, bronchoscopy has significant diagnostic limitations for pre-malignant lesions. These lesions are hard to detect visually because they are composed of a few layers of cells with a thickness of 0.2–1 mm and a diameter of a few millimeters.

It appears that the visualization or detection of these small squamous lesions requires a high level of training, as only 29% of the cases were detected by an experienced bronchoscopist. The development of fluorescence bronchoscopy addressed this limitation. However, although this method could localize early invasive and in situ cancers, the detection of dysplasia remained problematic. Furthermore, photodynamic diagnostic systems’ development was hampered by problems in sensitizing and interference with tissue autofluorescence. A new laser photodynamic diagnostic system was developed using tumor-specific drug fluorescence at 630 nm wavelength to overcome this. This wavelength is well separated from the typical endogenous fluorescence of the tissues, which ranges 500–580 nm.

Using a high-quality charge-coupled device and unique algorithm, the LIFE-lung Fluorescence Endoscopy was developed under the principle that dysplastic and malignant tissues reduce autofluorescence signals compared with normal tissues [33].

Several studies have been performed comparing the diagnostic specificity and sensitivity of LIFE bronchoscopy vs. WLB in diagnosing pre-invasive and early invasive lesions. Most of the studies reported a higher diagnostic sensitivity of LIFE bronchoscopy in detecting pre-malignant and early malignant lesions at the cost of lower specificity (i.e., more false-positive results). Surprisingly, the prevalence of pre-invasive and early lung cancer varies widely from one study to another. These fluctuations can be the result of the experience level of the operators.

Interestingly, the use of LIFE bronchoscopy identified a new morphological entity defined as Angiogenic Squamous Dysplasia (ASD). In a morphological study, angiodysplasia changes were frequently found in pre-neoplastic and early malignant lesions in the bronchi. This finding has been confirmed in pre-neoplasia among smokers. Thus, the significance of ASD for long-term follow-up and future studies evaluating the role of ASD as a biomarker for early lesions is an area of great interest [34,35,36,37].

## 7. Lung Tissue Biopsies

The gold standard for cancer confirmation is a biopsy of the tissues. Lung tissue biopsy samples must have adequate tissue material to identify the subtype of lung cancer by histopathology procedures. The initial biopsy is critical to confirm early diagnosis, avoiding repeating the biopsy with increased risk of complications and a delay in treatment initiation. Many commonly used procedures in diagnosing lung cancer include fiber optic bronchoscopy with or without transbronchial needle aspiration, endobronchial ultrasonography, image-guided trans-thoracic needle aspiration, mediastinoscopy, pleural fluid analysis (thoracentesis), thoracoscopy, and surgical approaches. These procedures are costly, prone to complications, and there is a possible need for more samples [36].

### Bridging between Traditional and New Screening Methods

The introduction of testing for biomarkers made the most use of lung tissue biopsies by testing for mutations. The most common targetable mutation in the gene *EGFR*, for which testing has been part of the standard practice since 2011, is not continuously assessed. It has been suggested that reflex testing can reduce the time to initiating treatment. Another setback is that tissue samples from biopsies are often sufficient for diagnosis but inadequate for biomarker testing, requiring recurrent biopsies, which might be challenging from a risk, cost, and patient preference standpoint. Tests may fail due to technical reasons. Proper diagnosis needs the collaboration of a multi-disciplinary system working with pulmonology or interventional radiology to ensure enough tissue is obtained at diagnosis for testing [20,37].

Oncomine (testing *EGFR*, *BRAF*, and *ROS1* only), MSK IMPACT (Integrated Mutation Profiling of Actionable Cancer Targets), and Foundation One CDx are FDA-approved next-generation sequencing (NGS)-based platforms for molecular testing. Broader NGS-based assays can evaluate emerging biomarkers in a single test to reduce the cost, test more patients, and reduce the need for recurrent biopsies.

One of the basic NGS assays commonly used in clinical molecular laboratories is the amplicon-based assay using multiple PCR primers to amplify genomic regions of interest directly. However, these assays have limitations in the number of genes and areas that can be effectively covered at once. As a result, these assays are typically small panels covering hot spots or highly selected clinical interest regions [38,39].

One of the critical issues facing practicing oncologists is whether to test tissue biopsy vs. liquid biopsy, primarily plasma-based circulating tumor DNA (ctDNA) assays, and whether ctDNA can now replace a biopsy or recurrent biopsies in some clinical settings [39,40]. However, this analysis is not practical for lung cancer diagnosis because mutations observed in cfDNA in peripheral blood do not necessarily coincide with the tumor-derived DNA from the same individual. Moreover, cfDNA can also be found in healthy individuals [41], making it a complicated task to differentiate cfDNA from non-tumor tissues. Although an alternative to circumvent this issue is the sequence of mutations in these cfDNA, non-tumor tissues might develop mutations that originated from the hematopoietic stem cells. These mutations originate from clonal hematopoiesis of indeterminate potential (CHIP) as a result of somatic mutations acquired with age [42].

Barriers to early lung cancer screening and diagnosis by traditional methods (discussed earlier) could be overcome with a simple, accurate, reproducible, and inexpensive yearly test as a general screening tool. Several biomarkers are emerging as tools for detecting early diagnosis. Because serologic biomarkers can be analyzed conveniently and economically, they are adequate for mass screening. Serologic biomarkers that are currently available for NSCLC are carcinoembryonic antigen (CEA) and serum cytokeratin 19 fragments (CYFRA 21-1) detailed below [43].

Peripheral biofluids, such as sera, are a preferred source of samples for identifying biomarkers for the early detection of tumors. Research has suggested that the early detection of lung cancer could be achieved by analyzing biomarkers in tissue samples from within the respiratory tract, including sputum, saliva, nasal/bronchial airway epithelial cells, and exhaled breath condensate. Moreover, the use of blood-borne biomarkers (liquid biopsies) includes circulating nucleic acids, proteins, and tumor cells (CTCs). Although the evaluation of these biomarkers requires a minimally invasive approach, it is also repeatable and inexpensive compared to imaging [44].

## 8. Transition to Biomarker Applications

In practice, the cornerstones of lung cancer assessment are radiology and tissue biopsies, as discussed earlier. Between missing early diagnosis, cost, and their risks, especially thoracic oncology biopsies, introducing the use of techniques as simple as a blood test provides a much safer and faster option. A review at the MD Anderson Cancer Center assessing cancer biopsies showed more than 17% adverse effects for thoracic biopsies [45].

### 8.1. cfDNA

This is a liquid biopsy that analyzes circulating free DNA (cfDNA) and CTCs via a non-invasive method, such as a routine blood draw or urine sample. cfDNA is released by normal cells and cells exhibiting pathologic processes (e.g., inflammation and neoplasia). Circulating tumor DNA (ctDNA) is a subset of cfDNA released by tumor cells that occurs through a combination of apoptosis, necrosis, and secretion. Analysis of the genetic alterations include point mutations, methylation patterns, chromosomal rearrangements, structural rearrangements, and copy number variations. Examples of cells contributing to cfDNA include the cells turning over due to: (i) Normal processes (e.g., lining of the gut), (ii) inflammatory events or other immune-mediated processes, and (iii) neoplastic phenomena. Thus, ctDNA is a tumor shed product. Normally, phagocytes clear cellular debris; however, this does not happen competently in solid tumors since cellular debris accumulates and is released into the blood [46]. Epigenetic screening is concerned with structural changes in chromosomal regions unrelated to DNA changes that mark altered activity states and show potential lung cancer diagnostic markers. DNA methylation and histone modification modulate gene expression that could influence early lung cancer detection [47].

Although the use of cfDNA showed promising results, further analyses of the published studies showed a different picture. For instance, a metanalysis comprised of 10 studies using cfDNA showed that a pool sensitivity of 0.8 was calculated with a range of 0.48 to 0.91 across the studies [48]. In the case of the specificity, a pool specificity of 0.77 was calculated, ranging between 0.47 to 1. Following this line of variation between the studies, an inconsistency of 86.6% and 93.4% was calculated by I-square [48]. These results signify that the pooled sensitivity and specificity of the studies are the result of heterogeneity rather than chance.

### 8.2. Blood Circulating Antigens

A number of antigens found in blood have been assessed over the years as potential biomarkers of lung cancer. The most studied biomarkers include CYFRA 21-1, carcinoembriogenic antigen (CEA), neuron specific enolase (NSE), and squamous cell carcinoma antigen (SCC-Ag). The following table is provided as an illustration of the sensitivities and specificities reported by clinical trials (Table 2).

As shown in Table 2, variations are observed across the different types of lung cancers. These variations could have originated in the cancer stage at the moment of blood collection and/or other methodologies used for the analysis, such as differences in the ELISA kits from different suppliers, including the threshold of the antigen values set by the company. Taken together, it appears that a unique antigen biomarker is not valuable for diagnostic and likely, a multi-antigen approach should be considered in combination or not with other biomarkers.

### 8.3. Cell-Free DNA (cfDNA) and Circulating Tumor Cells (CTCs)

The first discovery of circulating DNA and RNA in the plasma of healthy and sick individuals commenced in 1948 [55]. This discovery was later acknowledged more than 30 years later when increased amounts were found in cancer patients. Throughout the decade of 2000–2010, studies implying a direct relation between cfDNA and cancer found an increase in the tumor size and the quantity of cellular debris [55]. It was also found that cfDNA exists at stable levels with a concomitant increase due to cell injury. Hence, cfDNA was proposed as a marker of cancer cell death. Efforts to use cfDNA as a diagnostic and screening biomarker have been shown to identify early-stage lung cancer. The detection of ctDNA in plasma depends upon cfDNA shed, which is calculated via the difference in rates between the release of DNA by tumor cells vs. the renal clearance. Among the key variables are the mitotic rate and tumor. For example, it would favor ctDNA detection when metastasis, bone, or the liver is involved. On average, the amount of cfDNA found in a normal person ranges 5–10 ng/mL [55]. In cancer patients, depending on the type of cancer and stage, the cfDNA concentration might range up to 50 times more than the normal concentration.

Another way to investigate the cfDNA is by the use of the polymerase chain reaction (PCR). A study examined the levels of plasma DNA in 84 patients with NSCLC, which was compared to 43 healthy blood donor controls [56]. This study stated that healthy controls could be distinguished from patients with lung tumors. Even in patients with stage 1A, the amount of cfDNA in plasma was significantly higher than in the control patients. However, another study that also measured cfDNA as a screening tool found that cfDNA could not distinguish differences in a cohort of approximately 1000 high-risk smokers, suggesting that progression to lung cancer could not be predicted. Although cfDNA might not be an effective marker for screening high-risk smokers, it could still play a role in diagnosing whether nodules identified by LDCT are either benign or malignant. Studies have revealed limitations towards the advanced use of biomarkers in the clinic to facilitate physician adoption as part of their standard of care, mainly validity and maintaining general mass use of biomarkers in liquid biopsies [46,57,58,59].

Another predictor for cancer development is the quantification of CTC. CellSearch can perform this quantification. This platform uses whole blood to evaluate CTCs of epithelial origin in extensive clinical studies for breast and prostate cancer as markers of response to therapy and indicators of prognosis. Studies have revealed that CTCs in the blood are associated with a decrease in overall survival in patients treated for metastatic breast, colorectal, or prostate cancer. Thus, CTCs offer the opportunity to capture and profile individual aspects of a patient’s malignancy and have proven to be a vital cornerstone of precision medicine. Technical advances have now made it possible to detect and characterize single CTCs in the blood of patients. The identification of CTCs in the blood torrent platform measures the epithelial cell adhesion molecule (EpCAM) [60]. Non-EpCAM approaches for CTC capture and quantification are also under investigation. For instance, a further classification of CTCs using markers of transition from epithelial to mesenchymal markers could be used to monitor the progress of the disease.

## 9. Liquid Biopsies Use in Lung Cancer

The use of liquid biopsies could be in the form of introducing lung cancer genetic, transcriptomic, and epigenetic screening biomarkers to determine potential high-risk subjects as a preliminary screening before the use of CT. Thus, early diagnostic using biomarkers could diagnose intermediate nodules identified by CT, leading to selecting subjects that need a surgical biopsy and saving others who do not need it.

Liquid biopsies have clinical applications in early detection, tracking primary and metastatic foci, assessing and monitoring treatment, and treatment resistance. However, they come with a setback regarding mass implementation in that they require complicated analytical methods to analyze. Nevertheless, projects like the FDA Sequencing Quality Control Phase II (SEQC2) project and the Blood Profiling Atlas in Cancer (BloodPAC) consortium have focused on these aspects [55].

Liquid biopsies allow for the non-invasive analysis of body fluids for DNA-shed products and aberrant circulating cells. They have also been assessed as a pillar in the precision medicine field, as genetic analysis provides quantitative feedback and monitors patient responses, enabling a more precise, personalized, and practical approach towards individualized treatment.

The ability to use these non-invasive methods of analyzing liquid biopsies, such as plasma, saliva, pleural effusions, CSF, or urine at the clinic, is considered technological progress in immune-oncology.

Extensively studying and subtyping NSCLC through genetic analyses enables molecular understanding, resulting in more effective therapeutic options, significantly reducing toxicity profiles through target treatment of NSCLC subtypes (e.g., *EGFR*, *ALK*, and *ROS1*). Unfortunately, not many patients use target therapies. Nearly 80% of cancer patients do not have genetic mutation results available during the initial consult with an oncologist, and approximately25% begin cancer treatment before receiving results. Molecular diagnostic companies offer rapid services to overcome this issue, where whole blood is shipped overnight to identify ctDNA mutations (in *EGFR* and *KRAS*) using commercially available droplet digital PCR (ddPCR), and the results are reported within 72 h [61].

One of the challenges in using liquid biopsies to detect mutations arises from the difficulty of very low-frequency mutation detection. This might happen after lung cancer surgery for a curative intent (i.e., a small, localized tumor) as the source of ctDNA shed was removed, due to the continued clearance of ctDNA by the kidneys. In these cases, postoperative blood potentially requires the detection of mutations of ≤0.1%. Therefore, the improvement of specificity and sensitivity of lung biopsies is a field of technological research that could be used as adjuvant therapy and cancer screening.

## 10. Applications of Biomarkers in Clinical Samples

The clinical samples available for biomarker measurements are:

### 10.1. Sputum

Although cytological examination of sputum is a helpful screening tool for early diagnosis of lung cancer, peripheral tumors, such as adenocarcinomas arising from the smaller airways, can be missed.

PCR techniques have been used for the possible detection of molecular biomarkers for early lung cancer. This was highlighted in a study performed on 15 patients from a project called The Johns Hopkins Lung Project (JHLP) [31,62]. In this study, approximately 50% of the recruited patients (*n* = 15) with adenocarcinoma or large-cell carcinoma were detected by mutations in sputum cells before the clinical diagnosis (1–13 months) when traditional methods would have probably missed them.

Another gene of interest is the p16 gene, commonly inactivated or mutated in lung cancer [63]. The measurement of hypermethylation of the CpG islands in the sputum of lung cancer patients demonstrated a high correlation with early stages of NSCL cancer, suggesting that p16 CpG hypermethylation could be helpful in the early diagnosis of lung cancer.

The potential use of plasma microRNAs (miRNAs) as novel biomarkers for early detection of lung cancer has been studied. miRNA biomarkers also have the potential for lung cancer screening and early detection. These are non-coding RNAs with a length of 22 nucleotides targeting specific regions or mRNA sequences, usually found in the 3′ untranslated regions of mRNA, which either prevent translation or promote mRNA degradation and lead to down-regulation of particular genes. Being more stable than mRNA, miRNA used as a marker for lung cancer risk or diagnosis is more practical for clinical application.

Studies have shown that these miRNAs differentially circulate in plasma samples of lung cancer patients. These miRNAs include miR-155, miR-197, and miR-182, which have demonstrated high specificity and sensitivity to discriminate all cancer stages, including stage I of lung cancer, from cancer-free controls. Once validated in a large-scale clinical trial, these markers may be used as a non-invasive confirmatory screening test complementary to the LDCT screening procedure and used as a clinical test for monitoring and clinical follow-up of patients with lung cancer [61].

Several studies have explored the utility of miRNA-based biomarkers in sputum samples. miRNA profiles in the sputum could be used to identify NSCLC. More recently, studies were also able to identify and distinguish miRNA profiles that could be used in the early detection of SCC or adenocarcinoma. For example, an SCC signature of three miRNAs diagnosed the presence of a stage I SCC in patients’ sputum. The adenocarcinoma signature composed of four miRNAs detected no overlap between the two signatures in sputum in patients with stage I adenocarcinoma. Seven different miRNAs were identified in these two signatures, and these miRNAs could be used as risk factors for lung cancer. In bronchial tissue studies profiling miRNA in pre-malignant airway lesions, 69 miRNA were found to evolve in high-risk patients from a pre-invasive stage to a higher stage in the multistep process of lung carcinogenesis [64].

Although airway miRNA expression may serve as an early detection biomarker, it is limited to bronchial biopsies of pre-malignant airway lesions [65].

microRNA and methylome in plasma or urine.

### 10.2. Bronchoalveolar Lavage (BAL)

Routine cytopathological analysis of bronchoalveolar lavage (BAL) specimens has been used as a common diagnosis over the years. Currently, BAL is another specimen sample where the use of molecular biomarkers for early diagnosis is exploited. BAL involves the infusion and respiration of a sterile saline solution in distal segments of the lung via a fiberoptic bronchoscope. Molecular markers including *p53* mutations, *KRAS* mutation, the methylation status of the CpG island of the *p16* gene, and microsatellite alteration were studied in BAL samples. In addition, a study examined a series of 50 resected NSCLC tumor patients and compared the tumors and BALs concerning those molecular biomarkers. With the possible exception of the test for microsatellite alteration, all trials had relatively high sensitivity, detecting mutant cells in the presence of a significant excess of normal cells. The results showed that *p53* mutations were predominant in squamous cell tumors, whereas the *KRAS* mutations were predominant in adenocarcinoma tumors. Except for microsatellite alterations, the exact genetic change in the BAL sample as in tumors was always found. Unfortunately, small, peripherally located tumor results were the least specific, representing tumors where early intervention would be of great value. Further studies using these markers are necessary to apply to populations with increased risk, such as smokers without lung cancer and survivors of previous cancer [66].

### 10.3. Peripheral Blood

As discussed before, the limited accessibility of lung carcinomas has led to efforts to identify tumor-associated soluble markers in more accessible and non-invasive samples like serum or plasma. With the development of DNA technologies and the use of PCR techniques able to detect nanogram quantities of DNA circulating in the blood, it was found that the plasma and serum of cancer patients are enriched four times in DNA compared to free DNA from normal controls [67].

A comparison of microsatellite alterations in tumor and plasma DNAs was made in SCLC patients. Results showed that 93% of the patients with microsatellite alterations in tumor DNA also showed modifications in the plasma DNA [68]. These results suggest that modifications of circulating DNA can be used as an early detection biomarker. Another type of modification in circulating DNA is related to aberrant DNA methylation. The hypermethylated DNA was found in all cancer stages, opening up the possibility of an early lung cancer detection marker. Other gene mutations like *p53* and *RAS* gene mutations settled as markers in the plasma and serum of patients of other cancers like colorectal and pancreatic malignancies have not yet been established in lung cancer [47]. In addition, gene-expression alterations in circulating white blood cells have been identified in lung tumors.

Although the identification of a gene-expression biomarker in the blood is desirable by measuring mRNAs in the blood, studies have been relatively limited because of RNA degradation, restricting the use of blood-based transcriptomic biomarkers for early detection of lung cancer. For instance, a study analyzed gene expression in peripheral blood mononuclear cell samples of smokers with histologically diagnosed NSCLC tumors [69], and identified a signature of 29 genes that separate patients with and without lung cancer. Another study analyzed gene expression of lung tissue using serum RNA in whole peripheral blood collected using PAXgene blood RNA tubes. The study included patients with adenocarcinoma and controls to identify differential expression patterns of lung cancer genes that could be tested in blood to improve the identification of risky patients in the future. They showed that RNA-stabilized whole-blood samples could potentially be developed into a gene-expression-based classifier to discriminate between NSCLC patients and controls [67,70].

The stability of miRNA is attractive to explore because of its potential use as a blood biomarker for the early detection of lung cancer. Previous studies showed the applicability of miRNA in cancer diagnosis. For instance, a panel of seven miRNAs was differentially expressed in patients with cancer, as demonstrated by ultra-deep sequencing of blood samples from 10 patients with NSCLC and 10 healthy controls [71]. These results showed that miRNA signatures that predict lung cancer development and prognosis were identified.

When miRNA was analyzed in parallel to oncogene mutations, a better predictive occurrence of cancer was observed after finding a signature of six miRNA specific for lung cancer [72]. This finding is important because it connects a potential link between genetic damage and postgenomic control generated by the miRNA mechanism. This analysis is non-invasive, and both the miRNA and the oncogene mutations can be detected in the same sample. Moreover, deciphering oncogenic mutations, which might represent an individual signature, could be applied in the development of a personalized medicine in cancer prevention.

In summary, the use of miRNA is still questionable as more studies should demonstrate its applicability as a diagnosis of lung cancer.

The ELISA-based method for detecting the open reading frame 1 protein (ORF1p) in serum biomarkers can be used to identify patients at high risk of developing lung cancer based on LDCT findings. As such, ORF1p quantification in serum may provide a minimally invasive technique that can complement current lung cancer screening with LDCT [73].

### 10.4. Urine

Urine is seldom examined in the search for biomarkers. However, urine shows potential for use as a biomarker of lung cancer. Different analytes, such as a signature of volatile organic compounds (VOCs) and proteomic analyses, have been proposed as potential biomarkers for lung cancer diagnosis.

In the case of the VOC signature, it is expected that each individual would develop a unique signature. A study that aimed to assess the feasibility of VOC measurement to find biomarkers recruited patients with various lung cancer types. Urine samples were collected and analyzed using a urine cartridge sensor with an array of 73 spots [74]. Results showed that accuracies with sensitivities and specificities varied, with values of 36–95.5% and 60–97.6, respectively. These variations were obtained when different cancers were compared to the controls [74]. Although VOCs are very promising, more studies are necessary to validate the test for clinical. Moreover, it is clear that the VOC signature will fluctuate according to the phenotype of the individual as well as the diet and ethnic group. Thus, all these factors would have a direct effect on the baseline of the VOCs. Another limitation of the study is the sample size, but preliminary results are encouraging.

Another recently evaluated test is the conversion of the antiviral FDA-approved drug amantadine. It has been reported that the drug amantadine is acetylated by the enzyme spermine spermidine N^1^-acetyltransferase or SSAT-1 [75], and the levels of acetylated amantadine can be detected in urine samples as the acetylated product is not catalyzed anymore [76]. SSAT-1 is an enzyme upregulated in lung cancer, probably because of its function in the cell cycle [77]. The amantadine test is simple, and an individual is required to consume the amantadine pill and provide a urine sample (time = 0). After 2 h, a second urine sample is collected (time = 2 h). The measurement of acetyl amantadine levels in urine can be used to indicate the progress of cancer, and it is non-invasive (urine sample), simple, with clinical applicability for lung cancer diagnosis. For example, a receiver operating characteristic analysis showed an area under the curve = 0.689 when comparing lung cancer patients vs. healthy controls [76] in a clinical trial. Thus, the levels of acetylated amantadine could be used as a helpful and straightforward screening test for early diagnosis of lung cancer [75,78,79,80]. Other applications could be monitoring individuals working with carcinogenetic materials (e.g., asbestos), smokers, high-risk populations, and monitoring cancer recurrence after therapy.

Recently, the potential use of extracellular vesicles was proposed [81]. These vesicles were isolated from different body fluids, including urine, BAL, and serum of mice. The EpCAM levels were measured in these vesicles, as this protein has a role in tumorigenesis as a cell-cell adhesion mediator [82]. When mice were subjected to smoking, an increase in the vesicle concentration in BAL was observed, suggesting that the detection of vesicles in different body fluids might be potentially applicable for an early diagnosis of lung cancer. However, it is noteworthy that vesicles have not been tested in humans, and more studies should be performed for the validation of the test.

### 10.5. Metabolomics

Metabolomics data have the advantage of providing information on the levels of metabolites that can characterize the stage of the disease. Recently, the application of metabolomics for predicting cancer development has been reported in different fluids, such as serum, sputum, urine, and sweat, with promising results [83,84,85,86,87].

Metabolomic studies using serum showed that the discriminating metabolites aspartic acid and pyruvic acid differentiated individuals with lung cancer from healthy controls [84]. However, another study showed a different set of discriminating metabolites, such as glycerophospho-β-arachidonoyl ethanolamine and sphingosine with sensitivities and specificities of 77% and 93%, and 97% and 90%, respectively [86].

When sputum was analyzed to discriminate lung cancer patients from healthy controls, the metabolites cardiolipin (derivatives), hexanal, cysteic acid, and hydroxypyruvic acid were significant, with AUC ranging between 0.81–1.0 [83]. In sweat analysis, the trisaccharide MG (22:2), nonanedioic acid, and unidentified tetrahexose and trihexose showed sensitivities and specificities of 80% and 79%, which were calculated with a false-positive and -negative factor of approximately 20% [85].

Lastly, hypothesizing that the level of SSAT-1 increased in lung cancer and taking into consideration that this enzyme is involved in the polyamine metabolism [88], it is reasonable to assess the polyamine pathways fluctuations. Thus, a panel of six metabolites corresponding to the polyamine pathway discriminated lung cancer patients from healthy controls with an area under the curve (AUC) = 0.97 [87]. Interestingly, an AUC >0.9 was measured when liquid biopsies corresponding to the early stage of NSCLC were assessed using five metabolites. These data allowed for the differentiation of stages I and II from healthy controls [89].

Although metabolomics is an emergent field in cancer diagnosis, more studies should be implemented to validate its use consistently. Many factors might contribute to heterogenic results when comparing metabolomics in the same fluid. Some of the fluctuations might originate from the time of sampling, daily changes of the metabolites, methods used for the analysis, including variations in the commercial kits or in-house analysis, stage of the lung cancer, and the individual size of the study. Taken together, the validation of metabolomics requires confirmatory studies for its use in clinical diagnosis.

## 11. Conclusions and Future Venues

The early diagnosis of lung cancer remains a challenge because most of the available techniques and methodologies currently in use can detect cancer in advanced stages when treatment and a cure may not be efficient to control the disease. Thus, although significant progress happened over the last years, early diagnosis is still not accurate.

Lung cancer is mainly diagnosed by bronchoscopy and biopsies. In the case of bronchoscopy, it appears that the experience of the bronchoscopist is crucial for an accurate diagnosis. Although bronchoscopy is a minimally invasive technique with discomfort for the patients, complications can arise, especially if biopsies are taken from the suspicious tissue. Then, screening for early lung cancer development is required for an early therapy that can improve the outcome of the disease.

In recent years, the search for biomarkers in human fluids has been an attractive methodology that has progressed in the right way. For instance, studies have shown that sputum, blood, and urine samples can answer the demand for biomarkers. Most of the published biomarkers are detected by PCR, metabolomics, or by other molecular biology techniques that will provide fast results for early intervention. In addition, the use of urine samples has proven that the detection of a metabolite in lung cancer is feasible, and it can be performed in a matter of hours.

In summary, we believe that the trend in the development of more reliable tests for early diagnosis of lung cancer should be focused on biomarker discovery that will alleviate the discomfort of the patients, as well as the burden for the health authorities, as the techniques and methodologies currently in use are expensive.

## Figures and Tables

**Table 1:** TNM of malignant tumor classification.

Tumor Size (cm)		Lymph Nodes		Metastasis	
T1	<3	N0	No lymph nodes	M0	No
T2	T2a 3–5 T2b 5–7	N1	Ipsilateral bronchopulmonar/hilar lymph nodes	M1	Present
T3	>7	N2	Ipsilateral mediastinal/subcarinal lymph nodes		
T4	Invasion Mediastinal organs Vertebral bodies	N3	Contralateral/hilar/mediastinal supraclavicular lymph nodes		
Stage I			Stage II		
IA	T1, N0, M0		IIA	T2b, N0, M0	
IB	T2a, N0, M0		IIA	T1, N1, M0	
			IIA	T2a, N1, M0	
			IIB	T2b, N1, M0	
			IIB	T3, N0, M0	

**Table 2:** Sensitivity and specificity analysis of common antigens found in lung cancer.

Antigen Name	Type	Sensitivity (%)	Specificity (%)	Reference
CYFRA				
	SCLC	34	95	[49]
	NSCLC	49	95	[49]
	ND	43	89	[50]
	ND	85.1	88.3	[51]
	NSCLC	59	94	[52]
	SQC	68	94	[52]
	SCLC	19	94	[52]
	NSCLC	40	95	[53]
CEA				
	NSCLC	29	95	[49]
	ND	69	68	[50]
	ND	55	79.6	[51]
	NSCLC	42	95	[53]
SCC				
	NSCLC	17	95	[49]
	ND	35.6	71.2	[51]
	SQC	95	32	[54]
	NSCLC	19	95	[53]
NSE				
	SCLC	54	95	[49]
	ND	23.4	91.2	[51]

SCLC, Small-Cell Lung Cancer (SCLC); NSCLC, Non-Small-Cell Lung Cancer (NSCLC); SQC, squamous cell lung cancer; ND, not defined.
